# Bacterial Enhancer Binding Proteins—AAA^+^ Proteins in Transcription Activation

**DOI:** 10.3390/biom10030351

**Published:** 2020-02-25

**Authors:** Forson Gao, Amy E. Danson, Fuzhou Ye, Milija Jovanovic, Martin Buck, Xiaodong Zhang

**Affiliations:** 1 Section of Structural Biology, Department of Medicine, Imperial College London, London SW7 2AZ, UK; forson.gao15@imperial.ac.uk (F.G.); a.danson@imperial.ac.uk (A.E.D.); f.ye@imperial.ac.uk (F.Y.); 2 Department of Life Sciences, Imperial College London, London SW7 2AZ, UK; M.jovanovic@imperial.ac.uk (M.J.); m.buck@imperial.ac.uk (M.B.)

**Keywords:** gene transcription, AAA^+^, bacterial enhancer-binding protein, DNA opening, transcription initiation

## Abstract

Bacterial enhancer-binding proteins (bEBPs) are specialised transcriptional activators. bEBPs are hexameric AAA^+^ ATPases and use ATPase activities to remodel RNA polymerase (RNAP) complexes that contain the major variant sigma factor, σ^54^ to convert the initial closed complex to the transcription competent open complex. Earlier crystal structures of AAA^+^ domains alone have led to proposals of how nucleotide-bound states are sensed and propagated to substrate interactions. Recently, the structure of the AAA^+^ domain of a bEBP bound to RNAP-σ^54^-promoter DNA was revealed. Together with structures of the closed complex, an intermediate state where DNA is partially loaded into the RNAP cleft and the open promoter complex, a mechanistic understanding of how bEBPs use ATP to activate transcription can now be proposed. This review summarises current structural models and the emerging understanding of how this special class of AAA^+^ proteins utilises ATPase activities to allow σ^54^-dependent transcription initiation.

## 1. Introduction

Transcription initiation is the most regulated step of gene expression and is essential for the cells response to environmental changes [1]. Bacterial transcription initiation is regulated by a complex network of cell signalling pathways, which culminate in the recruitment of RNA polymerase (RNAP) to specific promoter regions by σ factors and the formation of open promoter complexes [2].

σ factors are directly responsible for promoter recognition, are the targets of transcription activator proteins and are required for DNA melting to make a transcription competent open promoter complex. There are two families of σ factor, based on sequence homology and the mechanism of action: σ^70^ and σ^54^, which are named after the molecular weights of the first members to be discovered. σ^70^ members recognise and bind to TATAAT consensus sequence motifs at the −10 region (upstream from the transcription start site at +1) and TTGACA consensus sequence at the −35 region. Upon binding to the promoter, the RNAP-σ^70^ holoenzyme forms a closed complex that can spontaneously isomerise to form an open promoter complex; for a full review, see the following citations [3,4,5].

In contrast, the σ^54^ family contains only one member, σ^54^ (also known as σ^N^), which binds to similar regions of RNAP, but has no discernible sequence homology and has significant differences in structure (with the exception of the helix-turn-helix motifs) and modes of RNAP regulation [3,6,7]. σ^54^ is present in an estimated 60% of bacterial genomes [8], and there are over 135 genes in *Escherichia coli* regulated by σ^54^ that cover a diverse range of stress responses [9], including nitrogen assimilation during starvation, response to antibiotics, carbon metabolism and loss of membrane integrity [10,11,12,13,14]. σ^54^ recognises the –12 (GG) and –24 (TGC) promoter regions and binds to the RNAP to form a stable closed complex that rarely spontaneously converts to open complex [15].

Transcriptionally competent open complex formation by the σ^54^ holoenzyme requires the actions of activators bound remotely upstream from the transcription start site. These activators, also called bacterial enhancer-binding proteins (bEBPs), belong to the AAA^+^ (ATPase associated with diverse range of cellular activities) family and ATP hydrolysis by bEBPs is required for the isomerisation from the closed complex to the open complex [16].

## 2. σ^54^ Domain Structure

**Figure 1:**
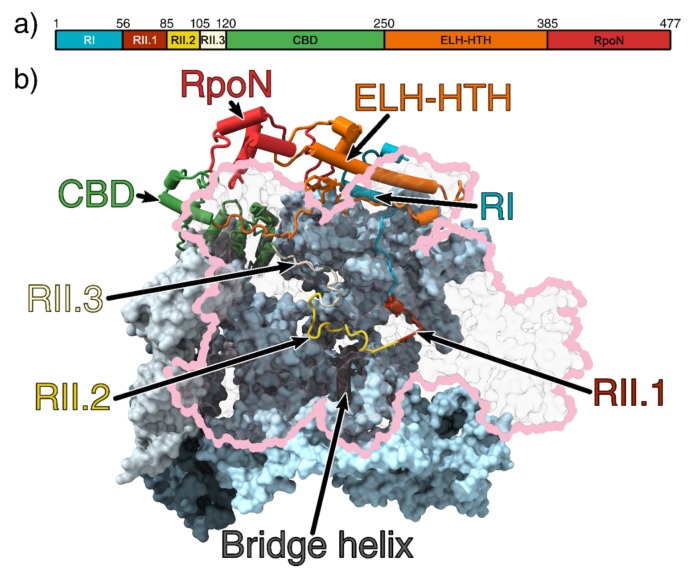
(**a**) Domain organisation of σ^54^. (**b**) The 3.8 Å crystal structure of the RNAP-σ^54^ holoenzyme (PDB ID: 5NWT) showing σ^54^ inside the RNAP cleft. Region I and an extra-long α-helix followed by a helix-turn-helix (ELH-HTH) sit outside the cleft to form a barrier to block DNA entry, whilst the core-binding domain (CBD) blocks the RNA exit channel. The β subunit is outlined in pink and transparent for clarity [6]. The catalytic site is made up of the β and β’ cleft (pink and light blue, respectively) and are stabilised by the α_1_ and α_2_ (light and dark grey, respectively) homodimers and ω subunit (obscured by the β’ subunit) [20,21]. Region I (RI) is coloured cyan; ELH-HTH, orange; RpoN, red; CBD, green; Region 2.1 (RII.1), crimson; RII.2, yellow; and RII.3, white. All figures rendered in ChimeraX [22].

σ^54^ is made up of three regions based on sequence conservation (referred to as RI-III) and four structural domains, which bind to RNAP to form the holoenzyme and interact with DNA (Figure 1a). Structures of the holoenzyme, the DNA bound closed complex (RPc) and the activator bound intermediate complex (RPi) reveal that Region I (RI) consists of two α-helices and, consistent with earlier biochemical data, is responsible for interaction with the bEBP and DNA at the −12 region. Region III (RIII) consists of an RNAP core-binding domain (CBD), an extra-long α-helix followed by a helix-turn-helix (ELH-HTH) domain and an RpoN box domain (Figure 1a) [15,17,18]. RIII binds to the −12 and −24 promoter regions via the ELH-HTH domain and RpoN box domain respectively. RI interacts with RIII ELH-HTH to form a structural domain that binds to the β and β’ cleft [6,19] (Figure 1b).

Region II (RII) is largely unstructured and its length varies between 30 and 110 residues long between species. In the *E. coli* RNAP-σ^54^ holoenzyme structure, RII is located inside the RNAP cleft, suggesting it could interfere with DNA entry and transcription bubble stabilisation. RII can be further subdivided into three regions (referred to as RII.1-RII.3) based on their location in the structure (Figure 1b). RII.1 is located where downstream DNA sits in the open and elongation complexes, close to the bridge helix at the catalytic centre, whilst RII.2 and RII.3 is located at the sites where DNA template strand and nascent RNA reside during transcription (Figure 1b) [6].

## 3. The Domain Architecture of bEBPs

bEBPs are often made of three domains: an N-terminal regulatory domain, which can be the receiver domain of a two component phospho-relay system (R), a central catalytic AAA^+^ domain (C) and a C-terminal DNA binding domain (D), although there are some bEBPs without R or D domains, with FleT being the only known bEBP that lacks both [23,24,25,26].

There are five different groups of bEBPs, categorised based on their biological functions and modes of regulation (Table 1) [27]. The R domain senses environmental signals and typically regulates the activity of the central AAA^+^ domain through either reinforcing or inhibiting hexamerisation, and/or inhibiting interaction with σ^54^. For a review on R domains and AAA^+^ domain regulation, see the following references [28,29,30,31].

The DNA binding domain (D) consists of the helix-turn-helix (HTH) motif that is part of either a 3 or 4-helix bundle, which is present in all groups except group 5 [27,28]. Typically, in the resting state, pairs of D domains bind to one or more upstream activating sequence (UAS) sites. The R domain typically serves as a constitutive inhibitor; upon receiving an activation signal inhibition is alleviated, and, with the help of integration host factor (IHF) to facilitate DNA looping, the AAA^+^ domain is brought in close proximity in order to interact with σ^54^, promoter DNA and the RNAP to activate transcription [29,32].

## 4. Conserved Motifs of the AAA^+^ (C) Domain

**Figure 2:**
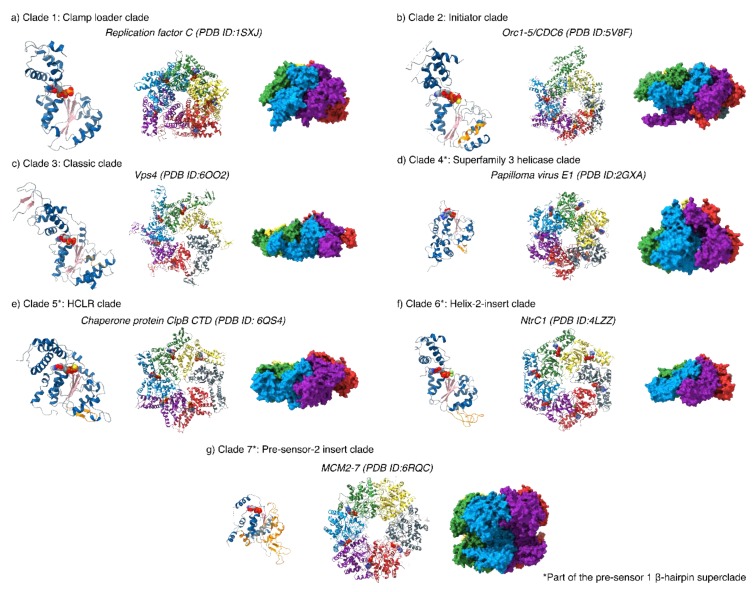
An overview of the different AAA^+^ protein clades. All AAA^+^ proteins form higher order homo- or hetero-oligomers that form asymmetric ring structures with heterogeneous nucleotide occupancies (28–33). All monomers contain a nucleotide binding site for ATP, and additional insertions (highlighted in orange on the monomer structures) that aid their unique functions. For monomeric structures: α-helices are coloured in blue; β-sheets, pink; insertions, orange and nucleotide and nucleotide analogues coloured by heteroatom.

**Figure 3:**
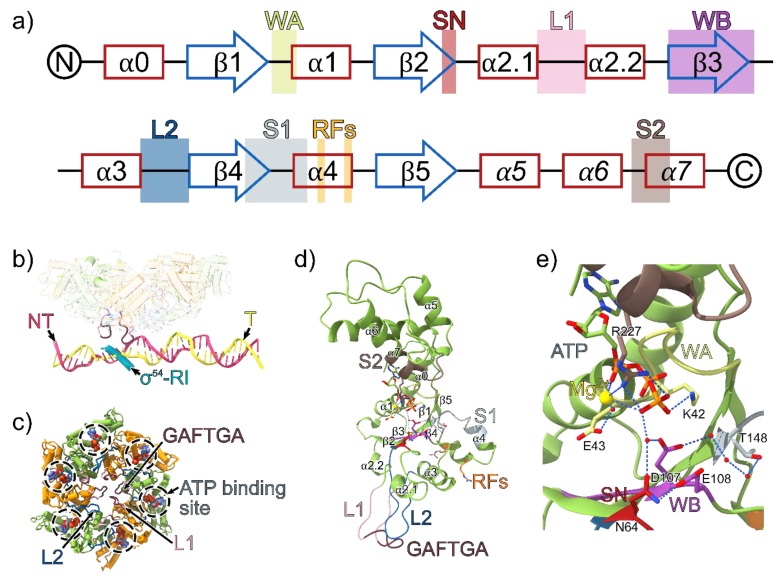
(**a**) The secondary structure of clade 6 AAA^+^ proteins with the positions of the conserved AAA^+^ motifs indicated. α helices are shown in red and β sheets are shown in blue. The α-helices in the lid subdomain are italicised. WA = Walker A, SN = Switch asparagine, L1 = Loop 1, WB = Walker B, L2 = Loop 2, S1 = sensor 1, RF = R fingers, S2 = Sensor 2. (**b**) The phage shock protein F (PspF) hexamer, σ^54^ region I (RI) and promoter DNA from the RPi structure (PDB ID: 5NSS). The highly conserved GAFTGA motif on the L1 loop projects downward from the plane of the PspF ring and contacts σ^54^-RI and the −11/−12 region on the promoter DNA. PspF monomers in the ring are coloured in green and orange; template strand (T), yellow; non-template strand (NT), pink; σ^54^-RI, cyan. (**c**) The PspF AAA^+^ domain hexamer from the viewpoint of the promoter DNA. ATP molecules are superimposed on the structure based on the ATP bound PspF crystal structure (PDB ID: 2C96). (**d**) PspF monomer model based on the crystal structure of an individual monomer of the AAA^+^ domain of PspF bound to ATP (PDB ID: 2C96). The solvent and magnesium atoms are superimposed from the ATP-bound PspF^R227A^ AAA^+^ domain (PDB ID: 2C9C), and the missing parts of the L1 and L2 loops are modelled from the ATP bound structure of NtrC1^E239A^ AAA^+^ domain (PDB ID: 3M0E). (**e**) The ATP binding site interactions within a PspF monomer.

The AAA^+^ domain of bEBPs is responsible for mechano-chemical coupling of ATP hydrolysis with remodelling of the RPc to enable transcriptional activation. bEBPs belong to the Helix-2-insert clade 6 of the AAA^+^ superfamily, which is part of the pre-sensor 1 β-hairpin superclade (clades 4–7); they differ from the classical clade 3 AAA^+^ protein in that it contains two additional loops [33]. These loops are referred to as loop 2 (L2), for the pre-sensor 1 β-hairpin that is characteristic of its superclade members, and loop 1 (L1), for the loop that is inserted in the middle of the α2 helix and is found in clade 6 (Figure 2 and Figure 3a,d). Interestingly, the L2 in bEBPs lacks the β-sheet secondary structure motifs and is thus more disordered compared to other members of the presensor-1 β-hairpin superclade. The classification of different AAA^+^ proteins is not only based on structural features, but also on biological function; certain members of clade 5, such as ClpB also contain insertions within the α2 helix that is referred to as a pore-1 loop, although clade 5 members serve different biological purposes to clade 6, primarily functioning as chaperones and protein translocases [33].

The L1/L2 loops directly interact with σ^54^ and the −12/−11 region of promoter DNA (Figure 3). The L1/L2 loops are candidates for delivery of one or more “power strokes” that trigger relocation of σ^54^ to allow for DNA melting and entry [34,35,36]. The L1 loop contains the highly conserved signature GAFTGA sequence motif in bEBPs. In most cases, GAFTGA mutants are unable to activate transcription, either due to the inability to inefficiently hydrolyse ATP, communicate nucleotide changes across the hexamer or interact with σ^54^ [28,37,38]. Conserved among AAA^+^ proteins, the nucleotide binding site is located in-between the α-lid and α/β sandwich subdomains of the AAA^+^ fold. Interesting, the canonical T/S residue after the lysine in the walker A motif (GXXXXGK[T/S]) are substituted for acidic D/E residues in bEBPs [28,39].

## 5. Roles of bEBPs in σ^54^-Mediated Transcription Initiation

**Figure 4:**
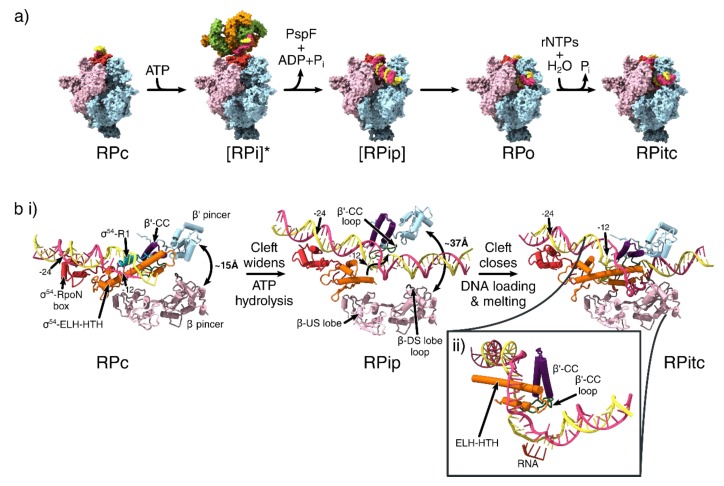
Conformational changes that occur during transcription initiation. (**a**) The overall structures of the different functional states of transcription initiation. View is from the downstream face. Colouring is identical to that of Figure 1b for β, β’, σ^54^ and α subunits and Figure 3c for PspF. [] = Transient intermediate. * = indicates the use of ADP.AlFx to capture the transient intermediate state. (**b**) DNA melting and clamp widening during initiation. Birds-eye view of cleft, looking down into the cleft, showing only the pincers, σ^54^ region I and III and DNA. The σ^54^- RpoN box is shown in red. σ^54^ Region I is not resolved in RPip and RPitc models. β-DS lobe loop shown in brown, β’-CC in purple and β’-CC loop in green. (ii) Side view of the transcription bubble in the RPitc structure. RNA shown in dark orange. (PDB IDs: 5NSR (RPc), 5NSS (RPi), 6GH6 (RPip), 6GH5 (RPo), 6GFW (RPitc)).

The cryo-electron microscopy (cryoEM) structures of the transcription closed (RPc), intermediate (RPi), partially loaded (RPip), open (RPo) and initially transcribing complexes (RPitc) have captured snapshots of the conformational changes during σ^54^ mediated and bEBP dependent transcription initiation (Figure 4a,b), explaining at multiple levels why the closed complex is unable to spontaneously proceed to an open complex and how bEBPs activate transcription by overcoming the strong transcription inhibition imposed by σ^54^.

The structure of RPc shows that the promoter DNA sat on top of the DNA binding cleft of the RNAP, with the σ^54^-ELH and RI forming a barrier preventing DNA from entering the cleft (Figure 4a). RI and the ELH-HTH were located at the −12 region, and significantly distorted and widened the minor groove downstream of this position (Figure 4b). Consistent with NMR [40] and crystal structures [6,19], the RpoN domain contacted the major groove of the −24 region using the conserved RpoN box residues from the same face of the promoter DNA as that of ELH-HTH. In support of their respective DNA binding roles, ELH-HTH Δ293-332 deletion mutants and RpoN R456 or R455 alanine mutants resulted in the inability to bind −12 and −24 regions of the promoter DNA respectively [40,41,42].

The σ^54^ CBD mainly contacts RNAP on the β’ subunit on the holoenzyme (Figure 1b), blocking the RNA exit site [6,15]. The inhibitory function of RI and ELH-HTH elements of σ^54^ has been corroborated by mutagenesis studies that led to the identification of activator bypass mutants. For example, deletion of RI resulted in the formation of stable open complexes in the absence of bEBPs, only with pre-melted DNA [43], but not with fully base-paired DNA [44]. Mutagenesis of residues 33–37 of RI allowed σ^54^ to initiate transient melting, but were unable to form a fully stable open complex [45]. Bypass mutants were also identified for three other ELH-HTH substitution mutations (most notably R336A), supporting the idea the ELH forms essential interactions with RI to initially prevent transcription [46].

The RPi structure revealed that the AAA^+^ domain of phage shock protein F (PspF) hexamer, in the presence of ATP hydrolysis transition state analogue ADP.AlFx (where x = 3 or 4), could engage with the RNAP-σ^54^-DNA complex via multiple AAA^+^ subunits (Figure 4a, second panel). It has been previously shown that RPi can synthesise short primed RNA in the presence of a partially opened transcription bubble, suggesting that this conformation represents an on-pathway intermediate state [47]. In accordance with previous low-resolution reconstructions, the PspF AAA^+^ hexamer is asymmetric and contacts the −12 region of DNA via its L1/L2 loops that descend downwards from the hexamer plane [36,48,49].

In comparison with RPc, the RI-ELH-HTH barrier moved upstream, the CBD moved away from the RNA exit site and the RNAP cleft was widened in RPi. This bEBP nucleotide binding state had therefore partially relieved the inhibition imposed by σ^54^. However, biochemical studies showed that ATP hydrolysis is required for complete transition to a transcription-competent open complex [50], and further conformational changes, promoted by ATP hydrolysis and ADP + P_i_ release, are required for the complete removal of the inhibition. In agreement with previous NMR studies, RI reached upwards to interact with the L1/L2 loops of bEBPs, and this interaction formed a wedge to separate the two DNA strands (Figure 3b), causing partial DNA melting of 5-6 bp (from −10 to −5), suggesting that bEBPs also played a role in DNA melting [15,51]. This is consistent with the fact that activator bypass mutants are unable to proceed to transcription with fully base-paired DNA [34].

These data strongly suggest that bEBPs activate transcription via at least three distinct roles: (1) interactions with σ^54^ cause conformational changes that remove the inhibition on RNAP imposed by σ^54^, (2) direct interactions with promoter DNA stabilise DNA distortions and therefore promotes transcription bubble formation and (3) interactions with σ^54^ form a structural wedge to separate DNA strands, thus also promoting and/or maintaining transcription bubble formation.

Once the inhibition by σ^54^ is released, DNA needs to be loaded into the cleft and the template strand delivered into the active site. Two structures provided mechanistic insights into this process. In one partially loaded state (RPip), the clamp was in a wide-open state with the ELH lowered into the cleft, thus enabling DNA, which interacted with ELH, to be loaded into the cleft (Figure 4a, third panel). The DNA had a significant 30° kink at the −10 to −5 region, resulting in DNA underwinding, which further assisted the melting of the DNA [34] (Figure 4b, second panel).

In the RPo structure, the RNAP cleft was once again in a closed conformation and the DNA had fully entered the cleft (Figure 4a), the ELH-HTH delineated the point of strand separation, inserting itself between the DNA to separate the two strands, creating a 13-nucleotide transcription bubble (from −11 to +2; Figure 4b, third panel), with the +1 of template strand positioned in the correct orientation for base-pairing with incoming ribonucleotides [34].

The observations of the wide-open RNAP cleft and lowering of ELH/DNA into the cleft in RPip as well as the narrower cleft and ELH inserted between the template and non-template strands in RPo, suggest that σ^54^-mediated transcription initiation occurs via two stages. The first stage involves cleft opening accompanied by initial DNA loading, while the second stage involves a coupled DNA loading and melting, which is accompanied by cleft closure. The binding of the bEBP and ATP hydrolysis releases the inhibition imposed by σ^54^ and promotes transcription bubble formation as bEBP-σ^54^ interactions and bEBP-DNA interactions are associated with DNA distortion observed in RPi. Subsequently σ^54^ facilitates DNA entry and stabilises the transcription bubble [34].

## 6. Models of ATP-Hydrolysis Coordination in bEBPs

In the absence of high-resolution structural information of RPi, especially snapshots during ATP hydrolysis, the precise mechanisms of how bEBPs utilise ATPase activity to remodel RPc are unknown. Recently a number of studies, largely based on cryoEM structures of AAA^+^ proteins with substrates bound in the central pores, have led to the proposal of a universal “hand-over-hand” mechanism of substrate threading coupled with ATPase activity within the hexamer [52]. However, given that bEBPs are not known to thread substrates through its pore and the clear lack of structural information implicating that σ^54^ or indeed DNA is threaded through the central pore, the conformational changes occurring within a bEBP hexamer during hydrolysis remain unclear.

Previous biochemical studies have found that communication between monomers in the bEBP ring is essential for transcription activation, and ATP hydrolysis by bEBPs is likely to operate via a sequential or at least partially sequential mechanism [53,54]. Based on crystal structures of bEBP without RNAP-σ^54^-DNA, two distinct but not mutually exclusive models were proposed for how nucleotide bound states are transmitted to enable coordinated L1/L2 loop movement [36]. All crystal structures were obtained by using either hydrolysis-resistant nucleotide analogues, hydrolysis-defective mutants or in the absence of magnesium [35,36,49].

Comparisons of the 1.9–2.1 Å resolution crystal structures of ATP-bound magnesium-deficient, ATP-bound arginine finger mutants and ADP-bound monomers of PspF lead to the proposal of the “glutamate-switch” model. In this model, the γ-phosphate is sensed by the Walker B (DExx) E108 residue, which forms interactions with the phosphate via a water molecule, and directly interacts with an asparagine residue in the ATP-bound state. The asparagine residue stabilises a network of hydrogen bonds that holds the L1 and L2 loops in a raised conformation to interact with the RNAP-σ^54^-DNA complex. Upon hydrolysis, the γ-phosphate is released and the glutamate pivots 90°, and can no longer interact with asparagine; this is thought to result in the release of the α2.1 and α2.2 helix containing the L1 loop (Figure 3a,c), enabling the movement of the L1 loop and remodelling of the RNAP-σ^54^-DNA complex. Due to the monomeric nature of the crystal structures, no information on the interaction between monomers in a functioning hexamer could be directly determined, and movement of the arginine finger motifs were relatively minimal [55].

An alternative “rigid body roll” model has been proposed by comparing heptameric 2.6–3.1 Å resolution crystal structures of ATP-, ADP.BeFx- and ADP-bound NtrC1 AAA+ domain E239A (analogous to the Walker B E108 in PspF) mutants. In the ATP-bound structure, the R299 finger forms two hydrogen bonds *in trans* with the γ-phosphate (R299 is analogous to R168 in PspF) and stabilises an essential lysine-glutamate interaction that holds the L1 loop in a raised conformation. Upon ATP hydrolysis there is a 90° rotation of the arginine finger motif, resulting in breakage of the lysine-glutamate interaction resulting in the power stroke. Additionally, there is a large rotation of the α/β subdomain towards the α-helical domain. Consequently, the monomer–monomer interface is altered, triggering the adjacent monomer to undergo ATP hydrolysis [36]. However, it has been shown that in the heptameric form, bEBPs are inactive [54].

More recent work from Nixon and co-workers has elucidated a wildtype NtrC1 asymmetric hexamer structure bound to ADP and ADP.BeFx. The sixth monomer interface contains a large gap without any nucleotide bound. Superimposition of each individual monomer within the asymmetric hexamer supports the notion of a rigid body roll, with the empty interface being the site of nucleotide exchange [49]. However, the rotation of the arginine finger between the ADP.BeFx bound and ADP bound monomers is smaller in comparison to the ATP and ADP bound monomers in the heptameric structure, suggesting there might be other residues responsible for triggering the conformational changes.

## 7. Summary and Future Perspectives

The recently determined cryoEM structures of bEBP-RNAP-σ^54^-DNA RPi complexes demonstrated the roles of bEBPs in transcription activation, where the AAA^+^ domain functioned via a currently unclear mechanism that is unlikely to involve substrate translocation through a central pore. The ATPase activity of bEBPs was responsible for alleviating the inhibition imposed by σ^54^ and initiating the formation of the transcription bubble, which was subsequently loaded into the RNAP cleft. The RPi structure, captured by using ATP hydrolysis transition state analogue, ADP.AlFx, revealed that inhibition was only partially relieved through bEBP binding. Indeed, biochemical data show that the complete release of inhibition leading to transcription activation requires ATP hydrolysis per se [56,57]. The conformational changes during ATP hydrolysis were most likely sensed and transmitted through residues that can sense the presence of γ-phosphate, such as the glutamate-switch and the sensor arginine residues although precisely how they sense and transmit both within the monomer and via interfacial events throughout the hexamer remains to be determined. bEBPs not only played crucial roles in releasing inhibition, they also display extensive interactions with promoter DNA, coinciding with extensive DNA distortions. Furthermore, interactions between bEBP and σ^54^ formed a wedge that separated the two DNA strands, thus directly contributing to strand separation. The AAA^+^ bEBPs therefore played multiple roles in activating transcription.

In order to understand the molecular details of these distinct roles, additional bEBP-bound intermediate transcription complexes and higher resolution structures will be required. Furthermore, it has been shown that UAS binding is essential for activation in many bEBPs [29,58]. How bEBP binding to the UAS influences their activity remains unknown so far as all the structural studies of bEBPs are in the absence of UAS. We have a good understanding of how phosphorylation on R domains activates NtrC1 and NtrC4 [29,30,59,60,61,62], and biochemical evidence of how NO-binding GAF domain inhibits the AAA^+^ domain of NorR from mutagenesis studies [63,64]. However, we still have limited structural insights on how other R domains transmit signals, either ligand or protein binding, to the AAA^+^ domains to activate transcription [28,65]. Future kinetic and structural work will enable us to resolve the mechanisms of these fascinating remodelling proteins and enable us to understand their diverse roles in biology.

## Figures and Tables

**Table 1:** An overview of the different groups of bEBPs. More information about clade 6 AAA^+^ proteins is discussed below and in Figure 2.

Group	Examples	R Domain	Mode of Regulation	C Domain	D Domain
1	NtrC, NtrC1, NtrC4, ZraR, DctD	RR/PRD	Phosphorylation from 2 component systems	Clade 6 AAA^+^	HTH
2	DmpR, AcoR, PheR, PrdR, XylR	PAS/V4R/ACT	Ligand binding	Clade 6 AAA^+^	HTH
3	NorR, NifA, FhlA	GAF	Ligand binding	Clade 6 AAA^+^	HTH
4	PspF, HrpR/S	N/A	Protein binding in *trans*	Clade 6 AAA^+^	HTH
5	FlgR, CtcC	RR	Phosphorylation from 2 component systems	Clade 6 AAA^+^	N/A

RR = Response regulator, PRD = Phosphotransferase System Regulation Domain, PAS = Per-ARNT-Sim domain, V4R = 4-vinyl reductase, ACT = aspartokinase, chorismate mutase, and TyrA, GAF = cGMP-specific phosphodiesterases, adenylyl cyclases and FhlA.
